# CLEAN AIR ACT 1990 AND ITS LONG-TERM IMPACT ON MORTALITY RISK AMONG OLDER ADULTS IN THE UNITED STATES

**DOI:** 10.1093/geroni/igae098.3119

**Published:** 2024-12-31

**Authors:** Siyao Lu, Emma Zang

**Affiliations:** Yale University, New Haven, Connecticut, United States; Yale University, New Haven, Connecticut, United States

## Abstract

**Background:**

Air pollution significantly influences public health, being a predominant cause of illness and death in the United States. The 1990 Clean Air Act (CAA) marks a pivotal regulatory initiative aimed at confronting this issue by enhancing air quality and public health outcomes. This study investigates the long-term effects of the CAA 1990 on mortality rates among the elderly, paying special attention to racial differences in health outcomes.

**Methods:**

This study adopts a difference-in-differences approach, analyzing county-level administrative data from 1982 to 2000. This approach evaluates the long-term impact of the CAA 1990’s implementation on the mortality rates of older adults across counties classified as “attainment” or “nonattainment” regarding the National Ambient Air Quality Standards (NAAQS) in 1990.

**Results:**

The analysis reveals that the CAA 1990 significantly lowered mortality risks among the older population. Black men experienced the most substantial health benefits, although the impacts on Black older adults manifested more slowly compared to their White counterparts. The next phase of this research will explore potential mechanisms by examining the CAA 1990’s influence on air pollution reduction.

**Discussion:**

This study underscores the CAA 1990’s role in mitigating public health burdens among older adults and highlights the essential need for policies that address racial disparities in the effects of environmental health. The insights are crucial for guiding future environmental policy, promoting environmental justice, and narrowing health disparities.

